# Factors associated with professional satisfaction in primary care: Results from EUprimecare project

**DOI:** 10.1080/13814788.2017.1305350

**Published:** 2017-04-10

**Authors:** Carlos Alberto Sanchez-Piedra, Lina Jaruseviciene, Francisco Javier Prado-Galbarro, Ida Liseckiene, Fernando Sánchez-Alonso, Sonia García-Pérez, Antonio Sarria Santamera

**Affiliations:** ^a^ Agency for Health Technology Assessment, Instituto de Salud Carlos IIIMadridSpain; ^b^ Rheumatology Spanish SocietyMadridSpain; ^c^ Lithuanian University of Health SciencesKaunasLithuania; ^d^ Red de Investigación en Servicios, Red de Servicios de Salud Orientados a Enfermedades Crónicas (REDISECC)MadridSpain; ^e^ Medicina Preventiva y Salud Pública, Universidad de Alcalá, ‘Alcalá de HenaresMadridSpain

**Keywords:** Primary health care, physician, primary care, consumer satisfaction, patient-centred care, quality indicators, quality of health care/standards, attitude of health personnel

## Abstract

**Background:** Given the importance of primary care to healthcare systems and population health, it seems crucial to identify factors that contribute to the quality of primary care. Professional satisfaction has been linked with quality of primary care. Physician dissatisfaction is considered a risk factor for burnout and leaving medicine.

**Objectives:** This study explored factors associated with professional satisfaction in seven European countries.

**Methods:** A survey was conducted among primary care physicians. Estonia, Finland, Germany and Hungary used a web-based survey, Italy and Lithuania a telephone survey, and Spain face to face interviews. Sociodemographic information (age, sex), professional experience and qualifications (years since graduation, years of experience in general practice), organizational variables related to primary care systems and satisfaction were included in the final version of the questionnaire. A logistic regression analysis was performed to assess the factors associated with satisfaction among physicians.

**Results:** A total of 1331 primary care physicians working in primary care services responded to the survey. More than half of the participants were satisfied with their work in primary care services (68.6%). We found significant associations between satisfaction and years of experience (OR = 1.01), integrated network of primary care centres (OR = 2.8), patients having direct access to specialists (OR = 1.3) and professionals having access to data on patient satisfaction (OR = 1.3). Public practice, rather than private practice, was associated with lower primary care professional satisfaction (OR = 0.8).

**Conclusion:** Elements related to the structure of primary care are associated with professional satisfaction. At the individual level, years of experience seems to be associated with higher professional satisfaction.

KEY MESSAGES

- A survey among primary care physicians conducted in seven European countries found that some organizational characteristics could explain professional satisfaction.
- Systems based on an integrated network and having direct access to specialists are factors associated with professional perception. Being a public sector employee presented a negative association with professional satisfaction.

## Introduction

Given the importance of primary care to healthcare systems and population health, it seems crucial to identify factors that contribute to the quality of primary care [1]. A recent review has found that the reduction of professional well-being and satisfaction could theoretically undermine their diligence, cognitive functioning, and relationships with patients [2]. Job satisfaction of primary care physicians has been linked with high performing primary care as well as, with significant patient satisfaction [3–6]. Chronic emotional and interpersonal stressors on the job produced suboptimal patient care and were considered potential risk factors for burnout and leaving medicine [7–12]. It is also important to identify and understand those factors as the attractiveness of primary care for young physicians depends on the job satisfaction of currently practicing general practitioners (GPs).

Research has also shown the association of organizational characteristics of primary care with satisfaction of primary care providers [13]. A survey performed in England revealed that salaried physicians compared to self-employed GPs, experienced resentment due to difference in status, decision-making autonomy and type of work they performed in practice [14]. However, salaried GPs reported lower stress compared with those GPs paid by mixed fee-for-service and capitation [15]. Although these findings implicitly indicate the potential influence that elements related to the structure of primary care could have on influencing professional satisfaction, large-scale empirical work providing international comparisons of primary care professional’s satisfaction in different primary care healthcare systems is scarce [16].

The EUprimecare project was conducted to assess quality and costs of different models of primary care systems in Europe. Germany, Spain, Estonia, Finland, Hungary, Italy, and Lithuania participated in this international project funded by the European Union [1,17,18]. As part of the project, a survey was conducted among primary care physicians with the aim of obtaining internationally comparable data on satisfaction with primary care in those seven countries. The aim of this article is to study physician satisfaction with primary care services, and to explore factors associated with satisfaction of primary care physicians in those countries.

## Methods

### Study sample and survey

This survey was planned to be completed by 176 primary care physicians per participating country (Estonia, Finland, Germany, Hungary, Italy, Lithuania, and Spain). A convenience sample size was obtained in each participating country. Inclusion criteria were GPs who work in primary care services. Physicians were selected according to a stratified sampling plan developed for each country considering either the list of all primary care practices/centres in a whole country (e.g. Estonia, Finland) or randomly selected primary care practices from certain municipalities or territories (e.g. Lithuania, Spain). Professionals were interviewed during October 2012. In total, 1331 questionnaires were completed. The methodology used to obtain the sample varied in each participating country, to ensure that the data collected being representative of the population the survey is intended to describe, a key element for guaranteeing the reliability of the study (Table 1).

**Table 1.** Survey methods and random sampling.

Country	Methods	Randomization
Spain	Face-to-face interviews	By visiting different practices, considering geographical representativeness.
Estonia	Web survey	The database of the primary care physicians was compiled considering geographical representativeness and they used random sampling.
Finland	Web survey	The database of the primary care physicians was compiled considering geographical representativeness and they used random sampling.
Italy	Computer assisted telephone interviewing (CATI)	Telextra business database which includes about 31 000 records, including phone numbers and other relevant contact information.
Germany	Web survey	The database of the primary care physicians was compiled considering geographical representativeness and they used random sampling.
Lithuania	Computer assisted telephone interviewing (CATI)	The database of the primary care physicians was compiled considering geographical representativeness and they used random sampling.
Hungary	Web survey	Database with physicians’ email addresses.

The study was approved by the ethics committee of the Instituto de Salud Carlos III.

#### Questionnaire

The questionnaire was developed by consensus among researchers from the EUprimecare partners. The dimensions of quality were defined through a process that started from focus groups with both patients and professionals. The questionnaire was designed in English. Afterwards, the questionnaire was translated into the language of the participating countries in EUprimecare using a dual focus method that sought to achieve conceptual as well as wording and grammatical equivalence. To ensure internal validity and comprehensible wording, the instrument was piloted on 10 primary care physicians in each country and refinements were made to the instrument.

### Variables

The questionnaire included variables exploring the following domains: sociodemographic data, organizational and financial aspects of the practice, activities conducted in primary care, satisfaction and burnout. We selected variables considering four domains based on Friedberg et al. [19]: personal effectiveness (receives and reviews data on patient satisfaction; years of experience; administrative activities); practice effectiveness (receives and reviews data on patient satisfaction; additional economic incentives; health centre which includes other professionals); relationships (patients have direct access to specialists; attend residents of a defined area); position within healthcare system (integrated network of health centres; patients have direct access to specialists).

### Statistical analysis

We developed descriptive analysis, using bivariate correlations and ordinal regression analysis to model the association between satisfaction and the possible explanatory variables. To evaluate professional satisfaction, we agreed to use the proxy variable ‘Overall satisfaction with your work in primary care: How satisfied are you?’ which could be answered on a scale from 1 to 5, meaning 1 very dissatisfied, and 5 totally dissatisfied. For this work, the values of this variable were recoded into two categories, low satisfaction (1, 2 and 3) and high satisfaction (4 and 5).

The independent variables were also items from the questionnaire. The following quantitative and qualitative characteristics were studied: country; variables related with the physician (age, sex, years since graduation, and years of experience in primary care); with the practice (urban or rural, single handed or group practice, primary care centre including other professionals; integrated network of primary care centres (permanent multidisciplinary teams including at least doctors and other health professionals which are functional and organizationally linked with other primary care centres to provide care to defined populations), patients having direct access to specialists, receiving and reviewing data on patient satisfaction; availability of electronic medical record, existence of additional economic incentives, who is the professional in charge of administrative tasks, and being a public sector employee).

First, bivariate logistic regression models were conducted to identify the effect of each independent variable on satisfaction. Second, multivariable analysis using logistic regression was used to establish the independent effect of professional characteristics and organizational features associated with GPs satisfaction with services provided in primary care, using only the variables that are statistically significant in the bivariate analysis. The model includes the odds ratio (OR) by the 95% confidence interval and statistical significance associated.

## Results

### Participants’ characteristics

A total of 1331 GPs working in primary care responded to the survey. Table 2 summarizes baseline characteristics including demographic, socio-economic and health characteristics depending on the level of satisfaction. The average age of respondents was 53.4 years (SD: 9.2). 68.6% of participants indicated they were satisfied with their work in primary care. Table 3 shows baseline characteristics and satisfaction depending on the country. The lowest level of satisfaction was found in Spain (18.8% declared being unsatisfied), whereas the highest level of satisfaction was found in Estonia, were 88.7% of patients claim to be highly satisfied.

**Table 2.** Participants’ characteristics according to level of professional satisfaction.

Variable	Highly satisfied	Poorly satisfied		*P*
Personal characteristics				
Gender, ref: male (%)	400 (43.81)	195 (46.65)	0.334	
Age, median (p25–p75)	54 (48–60)	54 (58–59)	0.733	
Years since graduation median (p25–p75)	29 (22–35)	29 (22–34)	0.917	
Years of experience in GP practice median (p25–p75)	24 (15–31)	22 (14–29)	0.006	
Organizational characteristics				
Work environment				
Single-handed (%)	376 (41.18)	208 (49.76)	0.003	
Group practice of GPs, ref: no. (%)	207 (22.67)	96 (22.97)	0.905	
Health centres, which include other professionals ref: no. (%)	255 (27.93)	102 (24.40)	0.178	
Attending residents of a defined area (%)	674 (73.82)	343 (82.06)	0.001	
Integrated network, ref: no. (%)	79 (8.65)	9 (2.15)	<0.001	
Nurses	243 (95.29)	97 (95.10)	0.937	
Pharmacists	51 (20.00)	19 (18.63)	0.768	
Nutritionists	48 (18.82)	18 (17.65)	0.796	
Social workers	143 (56.08)	65 (63.73)	0.186	
Psychologists	116 (45.49)	35 (34.31)	0.053	
Public/private service				
Public service (%)	418 (45.78)	225 (53.83)	0.006	
Private service (%)	405 (44.36)	142 (33.97)	<0.001	
Available resources				
Electronic medical record (%)	671 (73.49)	306 (73.21)	0.912	
Role of specialists				
Receiving clinical information from specialists (%)	615 (67.36)	244 (58.37)	0.001	
Patients having direct access to specialists (%)	322 (35.27)	108 (25.84)	0.001	
Geographic location				
Location, ref: urban (%)	689 (75.47)	311 (74.40)	0.677	

**Table 3.** Participants’ characteristics depending on country.

	Germany (*n* = 176)		Spain (*n* = 176)		Estonia (*n* = 176)		Finland (*n* = 198)		Hungary (*n* = 252)		Italy (*n* = 177)		Lithuania (*n* = 176)		
Variable	Median (p25–p75)	*n* (%)	Median (p25–p75)	*n* (%)	Median (p25–p75)	*n* (%)	Median (p25–p75)	*n* (%)	Median (p25–p75)	*n* (%)	Median (p25–p75)	*n* (%)	Median (p25–p75)	*n* (%)	*P*
Personal characteristics															
Age	53 (47–58)		54 (49–58)		53 (48.5–59)		55 (46–61)		57 (49–62.5)		54 (50–57)		54 (44.5–59)		0.042
Years since graduation	26.5 (19–32)		30 (24–34)		29 (24–34)		29 (20–35)		33 (24.5–38.5)		28 (24–32)		29.5 (19.5–35)		<0.001
Years of experience in GP practice	21 (12–26)		28 (21–32)		17 (14–22.50)		29 (20–35)		22 (13.50–30)		27 (22–30)		15 (12–29.50)		<0.001
Gender (ref: male)		112 (63.64)		76 (43.18)		9 (5.11)		100 (50.51)		131 (51.98)		145 (81.92)		22 (12.50)	<0.001
Organizational characteristics															
Practice type															
Single handed		94 (53.41)		165(93.75)		97 (55.11)		158 (79.80)		23 (9.13)		59 (33.33)		151 (85.80)	<0.001
Group practice of general practitioners (ref no.)		93 (52.84)		138 (78.41)		92 (52.27)		196 (98.99)		239 (94.84)		134 (75.71)		136 (77.27)	<0.001
Health centre which includes other professionals (ref no.)		166 (94.32)		44 (25)		162 (92.05)		131 (66.16)		243 (96.43)		167 (94.35)		61 (34.66)	<0.001
Integrated network (ref: no.)		176 (100)		176 (100)		176 (100)		123 (62.12)		252 (100)		167 (94.35)		173 (98.30)	<0.001
Nurses		5 (2.84)		131 (74.4)		12 (6.82)		65 (32.83)		7 (2.78)		8 (4.52)		112 (63.64)	
Pharmacists		1 (0.57)		5 (2.84)		6 (3.41)		11 (5.55)		4 (1.59)		0 (0)		43 (24.43)	
Nutritionists		4 (2.27)		4 (2.27)		3 (1.70)		20 (10.10)		5 (1.98)		1 (0.56)		29 (16.48)	
Social workers		3 (1.70)		115 (65.3)		2 (1.14)		26 (13.13)		2 (0.79)		1 (0.56)		59 (33.52)	
Psychologists		5 (2.84)		16 (9.09)		4 (2.27)		42 (21.21)		6 (2.38)		3 (1.69)		75 (42.61)	
Payment structure															
Public and private service		27 (15.34)		12 (6.82)		18 (10.23)		9 (4.55)		44 (17.46)		11 (6.21)		21 (11.93)	<0.001
Other elements															
Location (ref: urban)		121 (68.75)		152 (86.36)		121 (68.75)		170 (85.86)		169 (67.06)		112 (63.28)		155 (88.07)	<0.001
Attending residents of a defined area		131 (74.34)		170 (96.59)		112 (63.64)		87 (43.94)		241 (95.63)		147 (83.05)		129 (73.30)	<0.001
Overall satisfaction with your work in primary care															<0.001
Poorly satisfied		47 (26.70)		72 (40.91)		61 (34.66)		31 (15.66)		120 (47.62)		42 (23.73)		45 (25.57)	
Highly satisfied		129 (73.30)		104 (59.09)		115 (65.34)		167 (84.34)		132 (52.38)		135 (76.27)		131 (74.43)	
							

### Bivariate analyses

Bivariate analysis of the selected variables shows the relationship with overall satisfaction (Table 4). GPs satisfaction was positively associated with years of experience; for GPs working in an integrated network of primary care centres; GPs who receive and review data on patient satisfaction; and receive clinical information from specialists; and, when patients have direct access to specialists. The existence of group practice of GPs, health centres including other professionals, the availability of electronic medical records, the location of practice, additional economic incentives, and administrative activities were not associated with professional satisfaction.

**Table 4.** Variables associated with satisfaction in primary care: results of the bivariate analysis.

Variable	Wald	OR crude	95% CI	*P*
Sex (ref: female)	0.935	1.121	0.889–1.415	0.334
Single-handed or solo practice	8.537	0.707	0.560–0.892	0.003
Group practice of general practitioners/family physicians	0.014	0.983	0.747–1.295	0.905
Health centre which includes other professionals	1.186	1.201	0.920–1.566	0.178
Integrated network of health centres	16.723	4.305	2.138–8.665	<0.001
Public service	7.408	0.724	0.574–0.914	0.006
Patients having direct access to specialists	11.570	1.564	1.209–2.024	0.001
Receiving and reviews data on patient satisfaction	12.880	1.547	1.219–1.963	<0.001
Attending residents of a defined area	10.665	0.617	0.461–0.824	0.001
Electronic medical record	0.012	1.015	0.781–1.318	0.912
Location of your practice	0.174	1.058	0.811–1.381	0.677
Years of experience	3.195	1.011	1.000–1.023	0.048
Additional economic incentives	2.176	1.219	0.937–1.586	0.140
Receiving clinical information from specialists	10.070	1.472	1.159–1.868	0.002
Administrative activities	0.967	1.128	0.888–1.432	0.325

Dependent variable: job satisfaction (ref: high satisfaction).

OR: odds ratio; 95%CI: 95% confidence interval.

### Multivariable model

We studied the effect of the selected independent variables on overall satisfaction in a multivariable model. The reference category for the dependent variable of overall satisfaction was high satisfaction and all odds ratios (OR) were expressed in relation to this category. Table 5 presents the ordinal logistic regression results.

**Table 5.** Variables associated with satisfaction with primary care: Results of multivariate logistic regression analysis.

Variable	OR	95% CI
Sex (ref: female)	1.161	0.908–1.483
Single-handed or solo practice	0.811	0.627–1.050
Integrated network of health centres	2.832	1.360–5.894
Public service	0.757	0.588–0.973
Patients having direct access to specialists	1.303	1.004–1.719
Receiving and reviewing data on patient satisfaction	1.338	1.033–1.734
Attending residents of a defined area	0.802	0.588–1.093
Years of experience	1.013	1.001–1.025
Receiving clinical information from the specialists	1.223	0.943–1.586

OR: odds ratio; 95%CI: 95% confidence interval.

We found a positive and statistically significant effect of a higher probability of satisfaction in doctors working in primary care centres which are part of an integrated network (OR = 2.8, 95%CI: 1.4–5.9), when they receive and review data on patient satisfaction (OR = 1.3, 95%CI: 1.0–1.7), and with years of experience in primary care (OR = 1.0, 95%CI: 1.0–1.0), and when patients have direct access to specialists (OR = 1.3, 95%CI: 1.0–1.7). There was a negative and statistically significant effect of respondents that work as public employees as compared to being self-employed or being a private employee (OR = 0.8, 95%CI: 0.6–0.9).

## Discussion

### Main findings

In this work, we have studied the influence of organizational and individual characteristics on physician satisfaction with their work in primary care. One of the main findings of this work is that a large percentage of European GPs are highly satisfied with their work (68.6%), although there are significant differences in the level of satisfaction across countries.

These findings also suggest the significance of certain organizational factors of primary care in explaining variation in physician satisfaction beyond potential differences among countries. A third relevant finding is the influence of career stage in physician satisfaction.

### Interpretation of the study results

Overall job satisfaction is a question that has usually been applied for measuring physician job satisfaction since the work of Warr et al. [20]. It is the way satisfaction was explored in a recent reported published in the US [19]. The necessity to adapt to the complexity of primary care could explain the association with years of experience and satisfaction. This relationship is similar to the results of a study that found that professionals with less experience had the lowest satisfaction with overall career choice [21]. More complex models of care like integrated primary care centres have also shown to improve GP satisfaction. It could be hypothesized that in those clinical environments doctors perceive higher professional autonomy, work control and the opportunity to use their clinical abilities or inter-collaboration with other colleagues.

Interestingly, physicians who review information on their patient satisfaction are also more satisfied, as they could be in better position to ascertain whether they are meeting their patients’ needs.

We have found a negative association between overall satisfaction and working in public service. This relationship could be related to the hierarchical organizational culture of those health care establishments [22–24]. A study conducted in Lithuania came to a similar conclusion related to private and public healthcare sectors [25]. Then again, job control has been studied as one factor associated with physician satisfaction [26]. Our results suggest that more open, less rigid and controlled primary care services could lead to better results regarding GPs satisfaction. In this sense, the negative effect of gatekeeping function on primary care satisfaction seems to support this explanation.

Although previous research found an association of the availability of electronic medical records with satisfaction, our data did not identify this relationship [27,28].

### Strengths and limitations

Information about other factors that have been seen to influence GPs satisfaction, like time pressure or patient workload or type or complexity of patients seen, were not collected in the survey [29]. Other limitations of this study include the possible lack of representativeness of the sample. The study did not cover all European countries. The use of different methods to answer the survey in each country could be considered as a potential selection bias. It is also necessary to point out the possibility that at least some of the differences identified here between countries may be due to a different interpretation in each of the countries that participated in the study of the items that were collected in the questionnaire. The differences existing in primary care systems across Europe required developing specific methods to ensure the robustness of the data collected in each country. Variations in survey methodology may have affected findings such that within country differences could be valid but between country differences may be due to measurement effects.

Although our sample size was determined by convenience, the findings of an overall 68% of satisfaction indicate that with the sample size obtained, this study would be able to detect significant differences with a 90% confidence level and 5.7% margin of error.

## Conclusion

Surveying professionals’ attitudes regarding satisfaction with their job provides a valuable insight for improving primary care services. Understanding the determinants of primary care doctors’ satisfaction is relevant, because physicians who are satisfied are more likely to deliver better healthcare, as well as to improve patient satisfaction. It is therefore important to frame policies that enhance a work environment that could then lead not just to better results in terms of healthcare quality, but also to a higher attraction and retention of primary care doctors. Further studies should confirm the influence of cultural and individual factors, as well as the mechanisms that explain the association between gatekeeping and integrated network in GPs satisfaction.
